# Genetic association study of coronary collateral circulation in patients with coronary artery disease using 22 single nucleotide polymorphisms corresponding to 10 genes involved in postischemic neovascularization

**DOI:** 10.1186/s12872-015-0027-z

**Published:** 2015-05-12

**Authors:** Joan Duran, Pilar Sánchez Olavarría, Marina Mola, Víctor Götzens, Julio Carballo, Eva Martín Pelegrina, Màrius Petit, Omar Abdul-Jawad, Imanol Otaegui, Bruno García del Blanco, David García-Dorado, Josep Reig, Alex Cordero, Josep Maria de Anta

**Affiliations:** 1 Unitat d’Anatomia i Embriologia Humanes, Departament de Patologia i Terapèutica Experimental, Facultat de Medicina, Campus de Ciències de la Salut de Bellvitge, Universitat de Barcelona, L’Hospitalet de Llobregat, Barcelona, 08907 Spain; 2 Departamento de Estadística, Facultad de Ciencias, Universidad de Valparaíso, Valparaíso, Chile; 3 Neurovascular Research Group (NEUVAS), Institut Municipal d’Investigació Mèdica. Hospital del Mar, PRBB, Parc de Recerca Biomèdica de Barcelona, Barcelona, Spain; 4 Department of Cardiology and Hemodynamics, Centre Cardiovascular Sant Jordi, Barcelona, Spain; 5 Department of Cardiology, Hospital Universitari Vall d’Hebron, Barcelona, Spain; 6 Department of Cardiology, Hospital Mútua de Terrassa, Terrassa, Barcelona, Spain; 7 Departament of Morphological Sciences, Universitat Autònoma de Barcelona, Bellaterra, Cerdanyola del Vallès, Barcelona, Spain; 8 Àrea Epigenetics and Cancer Biology Area, Institut d’Investigació Biomèdica de Bellvitge (IDIBELL), L’Hospitalet de Llobregat, Barcelona, Spain

**Keywords:** Collateral circulation, Arteriogenesis, Postischemic neovascularization, Single nucleotide polymorphism, Genetic association study

## Abstract

**Background:**

Collateral growth in patients with coronary artery disease (CAD) is highly heterogeneous. Although multiple factors are thought to play a role in collateral development, the contribution of genetic factors to coronary collateral circulation (CCC) is largely unknown. The goal of this study was to assess whether functional single nucleotide polymorphisms (SNPs) in genes involved in vascular growth are associated with CCC.

**Methods:**

677 consecutive CAD patients were enrolled in the study and their CCC was assessed by the Rentrop method. 22 SNPs corresponding to 10 genes involved in postischemic neovascularization were genotyped and multivariate logistic regression models were adjusted using clinically relevant variables to estimate odds ratios and used to examine associations of allelic variants, genotypes and haplotypes with CCC.

**Results:**

Statistical analysis showed that the *HIF1A* rs11549465 and rs2057482; *VEGFA* rs2010963, rs1570360, rs699947, rs3025039 and rs833061; *KDR* rs1870377, rs2305948 and rs2071559; *CCL2* rs1024611, rs1024610, rs2857657 and rs2857654; *NOS3* rs1799983; *ICAM1* rs5498 and rs3093030; *TGFB1* rs1800469; *CD53* rs6679497; *POSTN* rs3829365 and rs1028728; and *LGALS2* rs7291467 polymorphisms, as well as their haplotype combinations, were not associated with CCC (p < 0.05).

**Conclusions:**

We could not validate in our cohort the association of the *NOS3* rs1799983, *HIF1A* rs11549465, *VEGFA* rs2010963 and rs699947, and *LGALS2* rs7291467 variants with CCC reported by other authors. A validated SNP-based genome-wide association study is required to identify polymorphisms influencing CCC.

## Background

In patients with coronary artery disease (CAD), the perfusion of the myocardial tissue is impaired. To mitigate myocardial ischemia, a neovascularization process, which includes the creation of a capillary network in the ischemic myocardium (angiogenesis) and the growth of collateral arteries (arteriogenesis) is initiated to enhance blood flow to the myocardium. Collateral arteries are natural vascular bypasses that can significantly reduce the degree of myocardial ischemia. They develop through the growth of small pre-existing arterioles [1]. Thus, patients with good collateral circulation have a lower mortality (36 %) than patients with low levels of collateralization [1]. Patients with CAD are highly heterogeneous in their arteriogenic response, even those with totally occluded arteries [2], with this variability attributed to genetic and environmental factors [3]. Collateral vascular growth and angiogenesis are parts of the same process leading to neovascularization. They complement each other: collateral growth and arteriogenesis provide bulk flow to the tissue, and angiogenesis promotes a capillary network that salvages the ischemic area. Angiogenesis and arteriogenesis are driven by distinct, but partially overlapping, cellular and molecular pathways [4]. In this study we examine putative genetic markers of coronary collateral growth. Our group has previously reported that the p.Pro141Leu polymorphism located in the urokinase-type plasminogen activator gene (*PLAU*), a gene expressed at collateral growth sites during arteriogenesis, is associated with coronary collateral development in patients with severe CAD [5]. To this end, we performed an association study to relate coronary collateral circulation (CCC) to 22 SNPs corresponding to 10 genes with suspected or demonstrated functional involvement in the process of postischemic neovascularization, and their corresponding haplotypes, in a Spanish cohort of patients with CAD.

## Methods

### Study subjects

The study was conducted between 2008 and 2012. We evaluated a Spanish cohort of 677 consecutive CAD patients with severe (≥70 %) stenosis who had been scheduled to undergo diagnostic coronary angiography at the Centre Cardiovascular Sant Jordi (CCSJ) or the Hospital Universitari Vall d’Hebron (HUVH) in Barcelona, Spain. The protocol was approved by the Bioethics Committee of the two centers (Ethics Committee of Clinical Research of the HUVH and the Bioethics Committee of the CCSJ), and authorized written consent was obtained from all the subjects. The exclusion criteria were: recent (less than 1 month previously) acute myocardial infarction; anemia; recent angioplasty; prior revascularization by percutaneous coronary intervention; coronary artery bypass surgery; and renal infection, inflammation or chronic failure. Epidemiological and clinical data included hypertension, diabetes mellitus (DM), DM type, hyperlipidemia, smoking history, family history of cardiopathies (FHC), history of angina, angina type and acute myocardial infarctions (AMI); with those not referring to type recorded as present or absent.

### Coronary angiography and coronary collateral artery scoring

Selective coronary angiography was performed using multiple orthogonal projections via the Judkins technique. Injection of the contrast in the donor artery was performed at a sustained high pressure with an automated controlled machine (ACIST CVi Contrast Delivery System®). CCC was assessed angiographically using a “modified” Rentrop’s method [6] without occlusion of the recipient artery. The following scale was used to assess the level of filling of the channels: 0 = no visible filling of any collateral channels; 1 = collateral filling of branches of the vessel to be dilated without any dye reaching the epicardial segment of that vessel (that is, right coronary artery injection showing retrograde filling of septal branches to their origin from the left anterior descending artery, without visualization of the latter occluded artery); 2 = partial collateral filling of the epicardial segment of the vessel being dilated; and 3 = complete collateral filling of the vessel being dilated. In patients with more than one collateral vessel, the highest Rentrop score was recorded.

CAD patients were classified according to the degree of CCC as either poor CCC (Rentrop 0–1) (n = 546) or good CCC (Rentrop 2–3) (n = 131). CCC was assessed by three experienced cardiologists who were blinded to the epidemiological, clinical and genetic data. The degree of agreement in the evaluation of CCC was high among the 3 observers, as determined by the kappa coefficient: κ = 0.987; 95 % confidence interval (95 %CI), 0.953-1.000 (P < 0.001) using the first 100 angiograms.

### SNP selection and genotype analysis

22 SNPs of genes involved in postischemic neovascularization were selected attending the following criteria: a) their suspected or proved functional or/and clinical significance regarding angiogenesis or arteriogenesis when known; b) their location within coding, 5' or 3' untranslated, or intronic sequences with known potential sites for factor binding; and c) a minor allele frequency of more than 5 % in the population studied (NCBI). We searched genes directly or indirectly involved in angiogenesis and/or arteriogenesis containing functional polymorphisms. Particularly, *HIF1A* [7–9], *VEGFA* [10–12], *KDR* [13, 14], *NOS3* [15, 16], *TGFB1* [17–19] and *LGALS2* [20, 21] have been involved in both processes. Furthermore, *CCL2* [22] and *ICAM1* [23] play an important role in arteriogenesis, while *CD53*, which controls TNFα levels [24], also plays an important role in this process [25]; and *POSTN* has been reported to be involved in angiogenesis [26] (Table 1). The SNPs located in or near these genes that were analyzed in this study are listed in Table 1 and details of them are as follows. *HIF1A* rs11549465 and rs2057482 affect mRNA production and are associated with CAD [27]; the first is also related to collateral circulation [28]*. VEGFA* rs2010963, rs1570360 and rs699947 influence protein production [29], and along with rs3025039 and rs833061 they have also been related to VEGFA serum levels [30–32]. Moreover, *VEGFA* rs2010963 and rs699947 have been associated with collateral circulation [33] and CAD [34]. *KDR* rs1870377 and rs2305948 affect primary protein structure, whereas rs2071559 is located 5’ upstream, being all related to CAD [35]. *CCL2* rs1024611 affects mRNA production [36–38]; and along with rs1024610, MCP1 plasma levels [39–41]. *CCL2* rs1024611 and rs1024610 have been associated with myocardial infarction [39, 42]. *NOS3* rs1799983 has functional consequences for the protein [43, 44] which are associated with coronary arteriogenesis [45, 46] and CAD [47]. *ICAM1* rs5498 affects the primary structure of the protein and both it and rs3093030, located near the 3’ end of the gene, are related to sICAM1 plasma levels [48–51] and to coronary artery calcification [52]. *TGFB1* rs1800469 is located towards the 5’ end of the gene and has been associated with coronary heart disease complications [53]. *CD53* rs6679497 is an intronic polymorphism associated with TNFα levels [24] which plays a role in modulating arteriogenesis [25]. *POSTN* rs3829365 and rs1028728 are located in the 5’ UTR of the gene, with the first being associated with heart failure [54]. Finally, *LGALS2* rs7291467 is located in intron 3 and has been associated with arteriogenesis [21] and CAD [55–57].

**Table 1:** **SNPs analyzed in the study**

Gene	Role in angiogenesis/arteriogenesis	SNP	Other HGVS names	Location	Functional category	FS score	Association to CCC	Association to CAD	Functional relevance
*HIF1A*	Both [7, 8]	rs11549465	p.Pro582Ser	Exon 2	Missense variant	0.627	[28]	[27]	Influences transactivation activity [27, 58]
		rs2057482	c.*45 T > C	3’-UTR	3’ UTR variant	0	-	[27]	Influences transactivation activity [27]
*VEGFA*	Both [10–12]	rs2010963	c.-634C > G	Promoter	Regulatory region variant	0.257	[33]	[34]	Influences protein production [29] Related to VEGFA serum levels [30]
		rs1570360	c.-1154A > G	Promoter	Regulatory region variant	0.242	-	-	Influences protein production and related to VEGFA serum levels [31]
		rs699947	c.-2055A > C	Upstream gene	Regulatory region variant	0.176	[33]	[34]	Influences protein production and related to VEGFA serum levels [30, 31]
		rs3025039	c.*237C > T	3’-UTR	3’ UTR variant	0	-	-	Related to VEGFA serum levels [32]
		rs833061	c.-958C > T	Promoter	Regulatory region variant	0.282	-	-	Related to VEGFA serum levels [30]
*KDR*	Both [13, 14]	rs1870377	p.Gln472His	Exon 11	Missense variant	0.103	-	[35]	-
		rs2305948	p.Val297Ile	Exon 7	Missense variant	0.621	-	[35]	-
		rs2071559	c.-906 T > C	Promoter flanking	Regulatory region variant		-	[35]	-
*CCL2*	Arteriogenesis [22]	rs1024611	g.2493A > G	Promoter flanking	Regulatory region variant	0.208	-	Related to myocardial infarction [39, 42]	Related to MCP1 serum levels [39–41] Influences mRNA expression [36–38]
		rs1024610	g.2936 T > A	Promoter flanking	Regulatory region variant	0.158	-	Related to myocardial infarction [39]	Related to MCP1 serum levels [39]
		rs2857657	g.5837G > C	Non coding exon	Non coding transcript exon variant	0.176	-	-	-
		rs2857654	g.2236C > A	Promoter flanking	Regulatory region variant	0	-	-	-
*NOS3*	Both [15, 16]	rs1799983	p.Asp298Glu	Exon 7	Missense variant	1	[45, 46]	[47]	Influences activity by different susceptibility to cleavage [43, 44]
*ICAM1*	Arteriogenesis [23]	rs5498	p.Lys469Glu	Exon 2	Missense variant	0.092	-	Related to coronary artery calcification [52]	Related to s-ICAM1 levels [48–50]
		rs3093030	c.-286C > T	Non coding exon	Non coding transcript exon variant	0.208	-	-	Related to s-ICAM1 levels [49, 51]
*TGFB1*	Both [17–19]	rs1800469	c.*309 T > C	Promoter	Regulatory region variant	0.208	-	[53]	-
*CD53*	-	rs6679497	c.-17-5027C > G	Intron 2	Regulatory region variant		-	-	Associated to TNFα levels [24], which has been related to arteriogenesis [25]
*POSTN*	Angiogenesis [26]	rs3829365	c.-33C > G	Promoter flanking	Regulatory region variant	0	-	Associated with heart failure [54]	-
		rs1028728	c.-953 T > A	Promoter flanking	Regulatory region variant	0.5	-	-	-
*LGALS2*	Both [20, 21]	rs7291467	c.6 + 3279C > T	Intron 1	Regulatory region variant		[21]	Related to myocardial infarction [55–57]	-

Abbreviations: CCC, coronary collateral circulation; CAD, coronary artery disease. FS score: functional effects of SNPs obtained from 16 bioinformatics tools and databases. (http://compbio.cs.queensu.ca/F-SNP/)

Blood samples were drawn from patients undergoing coronary artery catheterization. Genomic DNA was isolated using the QIAmp DNA Blood kit following the manufacturer’s protocol (Qiagen©, UK). TaqMan SNP genotyping assays (Applied Biosystems, Foster City, CA, USA) were performed to determine genotypes from the blood samples using a 7900HT Fast Real-Time PCR System (Applied Biosystems, Foster City, CA, USA). Genotype assessments were reproduced in three independent assays.

### Statistical Analysis

Data were summarized and presented in the form of mean, standard deviation and percentage as descriptive statistics. Continuous data that were not normal-distributed were analyzed using the Mann–Whitney *U* test. In this study, age does not show a normal distribution (Shapiro-Wilk p-value <0.001). Associations among categorical data were assessed using Fisher’s exact or chi-square test, and Hardy Weinberg equilibrium was assessed using the chi-square test. Multivariate logistic regression models were adjusted using clinically relevant variables to estimate odds ratios (ORs) and 95 %CIs among genotypes, haplotypes and the risk of poor CCC. Interaction terms between SNPs, haplotypes and significant covariates were also analyzed in the multivariate regression models. Statistical analysis was performed using STATA 11.2 software. The power to detect a genetic association was estimated using the same statistical package. The SNPStats software available at http://bioinfo.iconcologia.net/en/SNPStats_web was used to calculate linkage disequilibrium (measured as Lewontin’s D0-values) between SNPs, to estimate haplotype frequencies, and to evaluate haplotype association with CCC.

## Results

A total of 677 CAD patients (median of age 66 years, 107 females/570 males) stratified according to the level of coronary collateralization (546 poor; 131 good) were enrolled in the study. The clinical and epidemiological parameters of the patients according to CCC development are listed in Table 2. Statistical analysis showed that there were no differences among the poor and good CCC groups in terms of age, gender, hypertension or hyperlipidemia history, smoking, angina history or previous myocardial infarction (Table 2). However, the incidence of DM (55.9 %) and the percentage of patients prescribed with statins (44.3 %) were significantly higher in the poor CCC group, with p values of 0.037 and 0.035 respectively (Table 2).

**Table 2:** **Epidemiological and Clinical Characteristics of CAD patients with poor and good CCC**

Characteristic	Poor CCC	Good CCC	p value
	n = 546 (%)	n = 131 (%)	
Age *(years)*	65.26 ± 10.88	66.76 ± 10.06	0.187
Gender *(male)*	460 (84.25)	110 (83.97)	0.937
Hypertension *(n)*	372 (68.13)	97 (74.05)	0.188
Diabetes mellitus *(n)*	146 (26.74)	47 (35.88)	0.037*
Hyperlipidemia *(n)*	381 (69.78)	96 (73.28)	0.430
Smoking *(n)*	126 (23.08)	33 (25.19)	0.608
Angina history *(n)*	383 (70.15)	93 (70.99)	0.849
Previous myocardial infarction *(n)*	196 (35.90)	43 (32.82)	0.509
Medication with statins *(n)*	188 (34.43)	58 (44.27)	0.035*

Abbreviations: CCC, coronary collateral circulation. Values are given as mean (S.D.) or numbers of patients (%). p <0.05 was considered as statistically significant (*)

None of the SNPs studied, with the exception of *NOS3* rs1799983 and *POSTN* rs3829365, showed any deviation from Hardy–Weinberg equilibrium (HWE) (tested by conventional *χ*2) (Table 3). Therefore, rs1799983 (P_HWE__rs1799983_ = 0.0157) and rs3829365 (P_HWE__rs3829365_ = 0.0000) were not included in further genetic association tests.

**Table 3:** Association of genotype and allele distribution of examined polymorphisms with CAD patients with poor and good CCC

Gene	dbSNP ID	Patients	n	Genotype count (frequency)			P value^a^	Allele count (frequency)		P value^b^	HWE P
*VEGFA*	rs2010963			GG	GC	CC		G	C		
		Poor CCC	531	247 (46.52)	224 (42.18)	60 (11.30)	0.5760	718 (67.61)	344 (32.39)	0.495	0.8216
		Good CCC	121	50 (41.32)	58 (47.93)	13 (10.75)		158 (65.29)	84 (34.71)		
	rs1570360			GG	GA	AA		G	A		
		Poor CCC	451	207 (45.90)	197 (43.68)	47 (10.42)	0.782	611 (67.74)	291 (32.26)	0.521	0.8494
		Good CCC	97	47 (48.45)	42 (43.30)	8 (8.25)		136 (70.10)	58 (29.90)		
	rs699947			CC	AC	AA		C	A		
		Poor CCC	494	138 (27.94)	245 (49.59)	111 (22.47)	0.816	521 (52.73)	467 (47.27)	0.968	0.5199
		Good CCC	104	31 (29.81)	48 (46.15)	25 (24.04)		110 (52.90)	98 (47.12)		
	rs3025039			CC	CT	TT		C	T		
		Poor CCC	498	386 (77.51)	106 (21.29)	6 (1.20)	0.665	878 (88.15)	118 (11.85)	0.714	0.9533
		Good CCC	105	84 (80)	19 (18.10)	2 (1.90)		187 (89.05)	23 (10.95)		
	rs833061			CC	CT	TT		C	T		
		Poor CCC	526	124 (23.57)	268 (50.95)	134 (25.48)	0.471	516 (49.05)	536 (50.95)	0.232	0.6392
		Good CCC	121	33 (27.27)	63 (52.07)	25 (20.66)		129 (53.31)	113 (46.69)		
*KDR*	rs1870377			TT	AT	AA		T	A		
		Poor CCC	496	291 (58.67)	178 (35.89)	27 (5.44)	0.613	760 (76.61)	232 (23.39)	0.328	0.8991
		Good CCC	106	67 (63.21)	35 (33.02)	4 (3.77)		169 (79.72)	43 (20.28)		
	rs2305948			CC	CT	TT		C	T		
		Poor CCC	582	487 (83.68)	88 (15.12)	7 (1.20)	0.199	1062 (91.24)	102 (8.76)	0.207	0.3210
		Good CCC	153	120 (78.43)	32 (20.92)	1 (0.65)		272 (88.89)	34 (11.11)		
	rs2071559			TT	CT	CC		T	C		
		Poor CCC	544	147 (27.02)	276 (50.74)	121 (22.24)	0.319	570 (52.39)	518 (47.61)	0.140	0.8355
		Good CCC	129	29 (22.48)	64 (49.61)	36 (27.91)		122 (47.29)	136 (52.71)		
*CCL2*	rs1024611			AA	AG	GG		A	G		
		Poor CCC	576	332 (57.64)	210 (36.46)	34 (5.90)	0.221	874 (75.87)	278 (24.13)	0.826	0.3186
		Good CCC	153	94 (61.44)	46 (30.06)	13 (8.50)		234 (76.47)	72 (23.53)		
	rs1024610			AA	AT	TT		A	T		
		Poor CCC	516	312 (60.47)	180 (34.88)	24 (4.65)	0.516	804 (77.91)	228 (22.09)	0.715	0.6077
		Good CCC	112	68 (60.71)	36 (32.15)	8 (7.14)		172 (76.79)	52 (23.21)		
	rs2857657			CC	CG	GG		C	G		
		Poor CCC	511	309 (60.47)	181 (35.42)	21 (4.11)	0.365	799 (78.18)	223 (21.82)	0.832	0.8093
		Good CCC	111	71 (63.96)	33 (29.73)	7 (6.31)		175 (78.83)	47 (21.17)		
	rs2857654			CC	AC	AA		C	A		
		Poor CCC	580	336 (57.93)	211 (36.38)	33 (5.69)	0.248	883 (76.12)	277 (23.88)	0.993	0.4284
		Good CCC	153	93 (60.78)	47 (30.72)	13 (8.50)		233 (76.14)	73 (23.86)		
*NOS3*	rs1799983			GG	GT	TT		G	T		
		Poor CCC	513	211 (41.13)	216 (42.11)	86 (16.76)	0.596	638 (62.18)	388 (37.82)	0.686	0.0157*
		Good CCC	110	46 (41.82)	48 (43.64)	16 (14.54)		140 (63.64)	80 (36.36)		
*ICAM1*	rs5498			AA	AG	GG		A	G		
		Poor CCC	516	136 (26.36)	246 (47.67)	134 (25.97)	0.308	518 (50.19)	514 (49.81)	0.958	0.1039
		Good CCC	112	33 (29.46)	46 (41.08)	33 (29.46)		112 (50.00)	112 (50.00)		
	rs3093030			CC	CT	TT		C	T		
		Poor CCC	517	134 (25.92)	248 (47.97)	135 (26.11)	0.415	516 (49.90)	518 (50.10)	0.883	0.1535
		Good CCC	112	33 (29.46)	47 (41.97)	32 (28.57)		113 (50.45)	111 (49.55)		
*TGFB1*	rs1800469			GG	GA	AA		G	A		
		Poor CCC	483	198 (50.00)	228 (47.20)	57 (11.80)	0.696	624 (64.60)	342 (35.40)	0.979	0.8844
		Good CCC	100	43 (43.00)	43 (43.00)	14 (14.00)		129 (64.50)	71 (35.50)		
*CD53*	rs6679497			GG	GA	AA		G	A		
		Poor CCC	483	198 (41.00)	228 (47.20)	57 (11.80)	0.826	624 (64.60)	342 (35.40)	0.572	0.6712
		Good CCC	100	43 (43.00)	43 (43.00)	14 (14.00)		129 (64.50)	71 (35.50)		
*POSTN*	rs3829365			GG	GC	CC		G	C		
		Poor CCC	405	357 (88.15)	22 (5.43)	26 (6.42)	0.795	736 (90.86)	74 (9.14)	0.535	0.0000*
		Good CCC	76	69 (90.79)	3 (3.95)	4 (5.26)		141 (92.76)	11 (7.24)		
	rs1028728			AA	AT	TT		A	T		
		Poor CCC	389	242 (62.21)	128 (32.91)	19 (4.88)	0.230	612 (78.66)	166 (21.34)	0.105	0.7373
		Good CCC	77	54 (70.13)	22 (28.57)	1 (1.30)		130 (84.42)	24 (15.58)		
*LGALS2*	rs7291467			AA	AG	GG		A	G		
		Poor CCC	581	160 (27.54)	292 (50.26)	129 (22.20)	0.106	612 (52.67)	550 (47.33)	0.080	0.9589
		Good CCC	151	37 (24.50)	68 (45.03)	46 (30.47)		142 (47.02)	160 (52.98)		
*HIF1A*	rs11549465			CC	CT	TT		C	T		
		Poor CCC	518	402 (77.60)	111 (21.43)	5 (0.97)	0.563	915 (88.32)	121 (11.68)	0.474	0.4122
		Good CCC	112	84 (75)	26 (23.21)	2 (1.79)		194 (86.61)	30 (13.39)		
	rs2057482			CC	CT	TT		C	T		
		Poor CCC	497	339 (68.21)	148 (29.78)	10 (2.01)	0.490	826 (83.10)	168 (16.90)	0.328	0.1151
		Good CCC	111	70 (63.06)	38 (34.24)	3 (2.70)		178 (80.18)	44 (19.82)		

^a^Fisher’s exact test was used to evaluate differences between genotype groups. ^b^Pearson’s chi-squared, *χ*^2^, was used for to evaluate the allele distribution. *p <0.05 was considered as statistically significant

The genotype and allele distributions of all the polymorphisms in the population studied are shown in Table 2, and they did not show any differences between patients with good collateralization and patients with poor collateralization (p ≥0.05) (Table 3). Haplotype association analysis of polymorphisms in strong LD has more power than single locus tests to detect gene–disease associations. Thus, we also checked for haplotype combinations of polymorphisms in the *VEGFA*, *KDR*, *CCL2*, *ICAM1*, and *POSTN* genes to detect associations with CCC. To this end, we first estimated LD between the polymorphisms of these genes. There was a strong pairwise LD between the SNPs within these genes (data not shown), and *VEGFA*, *KDR*, *CCL2*, *ICAM1* and *POSTN* haplotype analysis showed that the haplotype frequencies in patients with good collaterals were similar to those in patients with poor CCC (data not shown).

## Discussion

An increasing number of SNPs are being accepted as underlying contributors to numerous cardiovascular disorders. Different researchers have shown the importance of several polymorphisms in CCC susceptibility [21, 28, 33, 46, 47]. *In vitro* studies have suggested that the p.Asp298Glu polymorphism plays a functional role, with the Asp 298 variant being associated with a decreased eNOS activity [43, 44], the consequences of which may include impaired collateral development. The Asp variant has been associated with poor CCC in 291 CAD patients with chronic coronary occlusions [45], and similar results have been reported in a series of 477 CAD patients with high-grade coronary stenosis ≥70 % [46]. However, because *NOS3* p.Asp298Glu deviates from HWE in our population, we could not analyze this polymorphism in our samples.

Another polymorphism which has been studied in relation to coronary arteriogenesis is p.Pro582Ser located in the *HIF1A* gene. The C/T polymorphism at nucleotide 85 of exon 12 results in a Pro/Ser polymorphism at residue 582 of HIF-1α. This substitution alters the amino acid sequence in the carboxyl-terminal domain of HIF-1α, which regulates protein stability and transcriptional activity [58]. Resar et al. demonstrated that CT or TT genotypes affecting residue 582 of the HIF-1α protein were associated with the absence of coronary collaterals in 100 patients with CAD [27]. This result indicates that p.Pro582Ser substitution could influence the expression of angiogenic growth factors, thus leading to reduced collateral formation. Although we could not validate these results in our 677 CAD patients, our results are in agreement with those published by Alidoosti et al. (2011) which found no association between rs11549465 variants and the extent of CCC (n = 196) [59]. Despite that study being conducted in Iranian CAD patients, our results support Alidoosti’s observations, with our study being more robust based on a significantly higher number of patients (n = 677). Taking all this into account, the relevance of p.Pro582Ser *HIF1A* to CCC susceptibility is still under debate.

Unlike the results reported by Lin et al., 2010, showing that the *VEGFA* c.-634C > G (+405C > G) (rs2010963) and c.-2055A > C (A-2578C) (rs699947) polymorphisms were associated with the coronary arteriogenic response in 393 CAD patients [33], our results do not confirm the existence of any association between CCC and the allelic or genotypic distribution of this polymorphism. Given that the study by Lin et al. was conducted in Chinese patients, this discrepancy could be attributed to differences in population genetics.

Galectin-2, which is encoded by the *LGALS2* gene, is an inhibitor of arteriogenesis [21]. This inhibition is dependent of the gene expression on the cell surface of monocytes, acting as a modulator of monocyte/macrophage responses during collateral artery growth. CAD patients with poor CCC have increased monocytic mRNA expression of galectin-2, independent of different stimulations of these cells. Interestingly, the mRNA expression of galectin-2 was significantly associated with the *LGALS2* rs7291467 genotype, which has been associated with CCC in a small group of patients (n = 50) [21]. The same researchers also found that galectin-2 was able to inhibit collateral circulation in a mouse model of limb ischemia [21]. However, we have being unable to demonstrate an association between arteriogenic response and the allelic or genotypic distribution of this polymorphism in our cohort of patients. This may be attributable to the fact that van der Laan’s study used the collateral flow index as a quantitative measure of CCC, instead of poor and good CCC based on a qualitative angiographic Rentrop score.

The most extensively studied chemokine contributing to postischemic neovascularization is the monocyte chemo-attractant protein-1 (MCP-1); a protein which is overexpressed in collateral growth, allowing for monocyte recruitment sites [60]. The crucial role of monocytes in collateral growth is exemplified by the observations that genetic targeting of the MCP-1 gene (*CCL2*) and of the MCP-1 receptor gene (*CCR2*) leads to defective collateral growth [61, 62]. However, none of the SNPs of *CCL2*, rs2857654, rs1024611, rs1024610 and rs2857657, analyzed individually or their haplotype combinations were associated with CCC development.

The main limitation of the study is that the collateralization assessment is based on the angiographic Rentrop score, which is a qualitative rather than a quantitative technique. A modified Rentrop method without occlusion of the recipient artery was performed in the current work. This method, as well as the inclusion of a large portion of patients with subocclusive lesions (>70-100 %), probably might explain why such a relative low number of patients displayed well-developed collateral arteries in this cohort. Also, functional polymorphims in interferon-beta signaling genes, which are involved in arteriogenesis from clinical studies [63, 64], were not included in the study.

## Conclusions

Despite having previously reported that *PLAU* p.Pro141Leu (rs2227564) was associated with coronary arteriogenesis [5], none of the rs11549465, rs2057482, rs2010963, rs1570360, rs699947, rs3025039, rs833061, rs1870377, rs2305948, rs2071559, rs1024611, rs1024610, rs2857657, rs2857654, rs1799983, rs5498, rs3093030, rs1800469, rs6679497, rs3829365 or rs1028728 polymorphisms analyzed located in or close to genes involved in postischemic neovascularization (*VEGFA*, *KDR*, *CCL2*, *ICAM1* and *POSTN*) or their haplotype combinations were associated with CCC development. In addition, in our cohort of patients we could not validate the association of the *NOS3* rs1799983, *HIF1A* rs11549465, *VEGFA* rs2010963 and rs699947, and *LGALS2* rs7291467 polymorphisms with CCC development reported by other authors. We and others have demonstrated the potential role of certain polymorphisms as factors associated with CCC [5, 21, 28, 45, 46], but usually they have not been validated in other cohorts of patients. In addition, SNPs may influence collateral development not only individually, but also when acting together with other SNPs, through gene haplotype networks, as demonstrated by the role of several inflammatory gene haplotype networks in CCC [65]. In conclusion, a validated SNP-based GWAS is needed to reveal and/or confirm the SNPs that predict coronary arteriogenic response.
